# Interaction Mechanism Characterized by Bond Performance and Diffusion Performance between TiO_2_@LDO and Asphalt Based on Molecular Dynamics Simulation

**DOI:** 10.3390/ma16227235

**Published:** 2023-11-20

**Authors:** Jinting Wu, Peirou Zhao, Ping Wang, Yang Guo, Fei Sun, Cheng Li

**Affiliations:** 1 College of Civil Engineering and Architecture, NingboTech University, Ningbo 315100, China; 2 College of Civil Engineering, Anhui Jianzhu University, Hefei 230601, China; 1310282453@std.ahjzu.edu.cn (P.Z.); 17764451128@163.com (F.S.); 3 College of Civil Engineering, Chongqing Jiaotong University, Chongqing 400074, China; 622220970146@mails.cqjtu.edu.cn (P.W.); 622210970193@mails.cqjtu.edu.cn (Y.G.); 622210970171@mails.cqjtu.edu.cn (C.L.)

**Keywords:** TiO_2_@LDO, asphalt, molecular dynamics, bonding, spread

## Abstract

In order to study the interaction between composite photocatalytic material TiO_2_@LDO and matrix asphalt, the four-component 12 molecular structure model of 70# matrix asphalt was optimized by using software Materials Studio 2020, and its heterostructure with TiO_2_@LDO composite was modeled. The bonding performance between asphalt and composite photocatalytic material was analyzed by interface energization, and the diffusion performance between asphalt and composite photocatalytic material was analyzed from the perspectives of particle movement and Z-direction density. By changing the temperature and other parameters in the simulation process, the change in bonding strength between TiO_2_@LDO and asphalt was investigated. Through the calculation and analysis of interaction energy, it was found that the adsorption and bonding strength between asphalt and TiO_2_@LDO were the strongest at 40 °C. At the same time, the diffusion performance was studied, and it was found that the molecular diffusion distribution of TiO_2_@LDO was more extensive at 60 °C, which laid the foundation for further blending of asphalt and TiO_2_@LDO. The simulation results show that TiO_2_@LDO molecules have a certain attraction to asphalt molecules and can modify the matrix asphalt to some extent.

## 1. Introduction

In recent years, China’s economic development level has gradually improved, and the number of cars has increased. Road traffic has brought a series of problems, while also improving production efficiency, ensuring economic development, and facilitating residents’ lives. According to a survey, nitrogen oxides (NO_x_), carbon monoxide (CO), and hydrocarbons (HC) in automobile exhaust pollutants in large cities in China account for 50%, 60%, and 30% of the total emissions, respectively [1]. Exhaust emissions not only bring pollution to the environment but also cause harm to human health, and exhaust emissions form acid rain after a series of reactions, leading to smog and air pollution [2]. Therefore, it is not enough to rely solely on the power of nature itself, and we need to take corresponding control and scientific governance measures. So, how to carry out green development has become a research focus. Among this, the use of photocatalytic technology to load road surfaces to degrade exhaust gas near road surfaces has become one of the hot spots in road-engineering research.

In Sendai, Japan, some researchers sprayed photocatalyst TiO_2_ on asphalt concrete pavement with large gaps and used it to degrade automobile exhaust on the pavement according to its photocatalytic oxidation–reduction reaction. Meng et al. think that more exploration of TiO_2_ should be based on photocatalysis. Although precious metals are now considered to be excellent cocatalysts, their practical application is still limited due to their noble costs [3]. Suárez found that TiO_2_ P25 led to the complete transformation of nitric oxide, but at the same time it also produced a lot of nitrogen dioxide [4]. Maggos et al. studied the removal of harmful gas NO_x_ in mortar, and found that its degradation effect on NO_x_ was very significant [5]. Etxeberria et al. found that nano-TiO_2_ sprayed on the surface still had a good NO_x_ removal rate even if it was partially covered with dust, and that its performance could be restored when it was washed with clean water; however, the impregnation of gasoline and other materials caused it to greatly lose its catalytic effect, and alkaline detergent or n-hexane solvent use permanently eliminated part of its degradation performance [6]. Jin et al. studied titanium dioxide montmorillonite (MMT) nanocomposites and found that anatase TiO_2_ nanomaterials can be embedded in the layer structure of MMT and dispersed on the outer surface; furthermore, they found that it can degrade the mixed polluting gases of NO, HC, and CO to some extent, enhancing its photocatalytic activity [7]. Dalton et al. used TiO_2_ for photocatalytic oxidation and reduction of NO_x_ gas, and it was found that TiO_2_ could effectively convert nitrogen oxides into harmless nitrates [8]. More and more scholars have studied TiO_2_, which has also attracted attention to the road.

It is worth noting that although TiO_2_ has a certain effect on the degradation of nitrogen oxides, the degradation efficiency of its traditional TiO_2_ photocatalytic materials is not high. Furthermore, the nitrate generated after the reaction, although harmless, will be washed into the soil by rain, which will cause soil eutrophication; this increase in nitrogen content will cause secondary pollution to the ecological environment of surrounding land and water, which has also become a bottleneck of the application of TiO_2_ photocatalytic materials. Layered double hydroxides (LDHs), also known as anion or hydrotalcite-like clay, represent a kind of layered compound composed of several metal elements. They are composed of a brucite main layer (positively charged) and a hydration exchange anion located in interlayer corridor for charge balance. Compared with cationic clay, an LDH matrix can be easily prepared in the laboratory. LDHs are exchangeable between layers of anions, and have been widely used in catalysis, adsorption, ion exchange, and other fields. Todorova et al. prepared a composite photocatalytic material of layered bimetallic oxide and TiO_2_ with a weight ratio of 1:1 to remove nitrogen oxides (NO_x_) under ultraviolet light and visible light, respectively. The experimental results show that the removal rate of nitrogen oxides by the TiO_2_ catalyst was significantly higher than that by the composite photocatalytic material under ultraviolet light and visible light, which was due to the synergistic effect between the composite materials [9]. Wang et al. prepared TiO_2_@LDO nano-composite photocatalytic material and studied the degradation of toluene under simulated illumination and real illumination. The results showed that both illuminations showed extremely-high photocatalytic activity for the degradation rate, which indicated that the TiO_2_@LDO composite material showed satisfactory performance in eliminating toluene in the air [10]. Dong et al. also studied the degradation of toluene by TiO_2_/Mg-Al LDH, and found that under light irradiation, the hydroxyl group at the edge of the LDH would lose electrons and be oxidized into hydroxyl radicals which then participated in the toluene reaction; they thought that this could provide an efficient and safe air purification strategy for the decomposition of harmful volatile organic compounds (VOCs) [11].

Although many scholars have studied the efficiency of the composite material TiO_2_@LDO, it has not been applied to asphalt materials, and how to evaluate composite photocatalytic asphalt materials with adsorption and photocatalysis synergy is a research difficulty. Therefore, it is worth studying by compounding TiO_2_ and LDHs to construct a heterojunction and use it in the construction of photocatalytic pavement.

Alternatively, with the progress of the times and the development of scientific analysis technology, more and more scholars began to combine molecular dynamics simulation (MD) with asphalt. In 1967, Dickie et al. studied asphalt fractions by various physical methods, and put forward a classical model of a colloidal system based on asphaltene multi-scale molecular aggregates [12]. According to the many years of research on asphaltene molecules, more scholars began to use the average molecular structure to characterize the molecular structure of asphalt, among which Jennings et al. put forward the strategic highway research plan and the average molecular structure of eight typical asphalts of A-355 [13]. Then, since 2007, some real asphalt models have been put forward, one after another. Zhang et al [14,15] proposed two three-component asphalt models for the first time, which can be applied to molecular dynamics simulation. Greenfield et al. developed the latest four-component molecular model of asphalt, including 12 molecules [15,16,17,18,19,20,21]. The four component 12-molecule asphalt structure model proposed by Li and Greenfield in 2014 has been used in many studies [20]. The four-component 12-molecule asphalt structure model proposed in 2014 is the most classic, and the AAA-1 asphalt model, which represents SHRP, is one of the most advanced asphalt models in the field of asphalt molecular simulation.

Molecular dynamics simulation can not only study the interaction between asphalt molecules and the properties of asphalt itself more intuitively, but also study the interaction between asphalt and aggregates, and between asphalt and photocatalytic materials [22]. Horgnies et al. studied the adhesion between aggregate and asphalt [23]. Ozkahraman pointed out that the performance of binder molecules is influenced by the chemical and mineral composition of aggregates [24]. Cao et al. studied the influence of TiO_2_ classical photocatalyst modification on asphalt, and the interaction between asphalt and photocatalyst was analyzed from macro to micro dimensions. The research shows that the interaction between TiO_2_ and asphalt is more significant, and that TiO_2_ becomes more closely connected with asphalt molecules and the free volume distribution becomes smaller [25].

**Figure 1:**
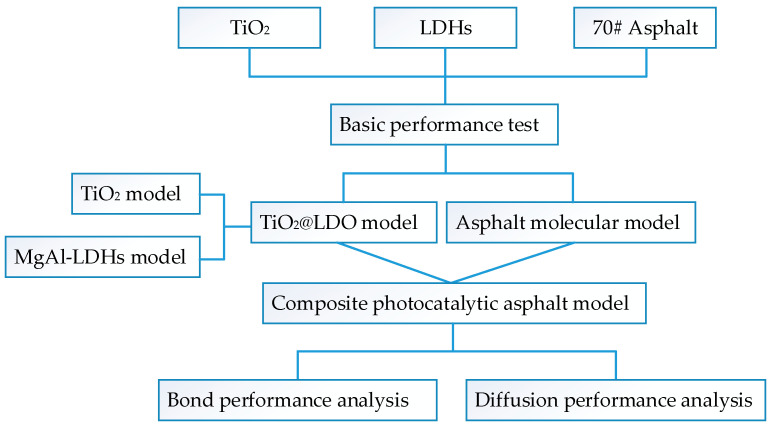
Research framework.

In this paper, in order to study the interaction between the composite photocatalytic material TiO_2_@LDO and matrix asphalt, MS molecular dynamics simulation software was used to study the bonding strength and diffusion performance of a photocatalytic asphalt binder, the model of TiO_2_@LDO and asphalt molecules was built by using the software, and the heterojunction structure was constructed. The interaction between asphalt and photocatalytic materials was investigated at four temperatures (0 °C, 20 °C, 40 °C, and 60 °C) by modeling, which provided theoretical basis for the better application of photocatalytic materials in asphalt. The comprehensive research framework is shown in Figure 1.

## 2. Materials and Methods

The test material involved in this paper, asphalt binder, adopts 70# matrix asphalt made in Ningbo China, and its basic performance parameters are shown in Table 1. There are three common allotropes of TiO_2_ made in Hebei China, namely, plate titanium type, rutile type, and anatase type, and their properties are different. Existing research shows that anatase TiO_2_ is more stable in the field of photocatalysis, so it is widely used. Therefore, anatase TiO_2_ was used in the selection of this model and the subsequent composite model. The basic performance parameters are shown in Table 2.

Mg-Al LDHs made in Guangzhou China, was used and its basic performance parameters are shown in Table 3.

Molecular dynamics simulation adopted in this paper was used to simulate the behavior of molecules under different conditions [26,27]. Molecules and atoms interact in a given period of time, and the trajectories of atoms and molecules are determined by Newton’s law, while the force field between atoms and molecules is defined by the applied force field. This method originated in 1950s and has been widely used in chemistry, physics, materials science, and biomolecules [28,29].

Molecular dynamics simulations can not only establish a three-dimensional molecular model more conveniently, but also avoid the material failure mechanism caused by dislocation of nanoparticles and molecular bond fracture, which may often be ignored. As such, this is a favorable study to explore micro-interactions and mechanisms. Molecular dynamics also provides a new way to analyze and understand materials from a specific angle, and can explore the behavior mechanisms of different materials, which provides a theoretical basis for researchers to modify and improve the properties of materials in the future. Moreover, molecular dynamics, as a kind of computer virtual-simulation experiment, is not limited by the number of samples and detection conditions and can overcome the macro shortcomings of an experiment to a greater extent. It can also reveal more micro mechanisms of macro experiments, combine macro and micro, greatly reduce the research cost, and provide the necessary theoretical basis for the experiment [2]. Microscopically, with the help of simulation technology, molecular structures, three-dimensional models, atomic or molecular trajectories, and some thermodynamic parameters can also be obtained, which provides strong support for the optimization and development of materials.

In this paper, MS 2020 (Material Studio) software was used for molecular dynamics simulation. MS simulation begins with the construction of a molecular model, and the atoms in each molecule are assigned a force field [22]. The main function of the force field is to determine the potential energy of the model molecule. After the force field, the periodic boundary conditions are defined, and then the appropriate ensemble is selected, and the model energy is minimized. There are many simulation modules in MS software, so we could select the appropriate module to simulate the simulated object and obtain the results needed by the experiment.

## 3. Establishment of Asphalt Molecular Model

The chemical structure and composition of asphalt components are very complex, involving thousands of molecules, and it is difficult to obtain detailed information about their chemical components. Because of this complexity, it is difficult to describe specific characteristics by using a single asphalt molecular average model, so it is necessary to determine appropriate models and corresponding parameters for the smooth follow-up research [30].

### 3.1. Molecular Model Selection

**Figure 2:**
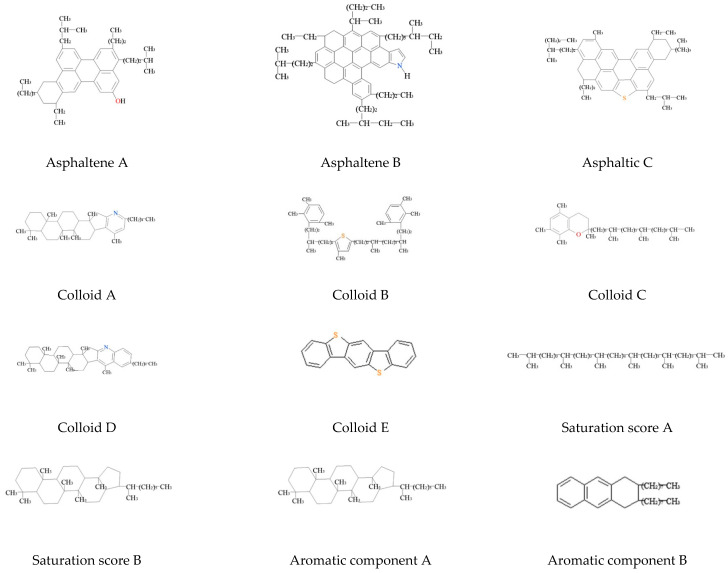
Molecular chemical structure diagram of asphalt components.

The twelve-component molecular model of asphalt was proven to be able to study the rheological characteristics, diffusion behavior, and adhesion with mineral powder of asphalt mortar. In this paper, the four-molecule twelve-component molecular model of asphalt, namely the SHRP AAA-1 asphalt model based on SARA, was selected [20,31,32]. The model is based on four components obtained from Corbett, ref. [33] including two molecular components of saturated component, two molecular components of aromatic component, three molecular components of asphaltene, and five molecular components of gum. The specific steps for constructing a molecular model refer to Zhu Jianyong’s research [34]. The molecular structural characteristics of the asphalt component are shown in Table 4, and the molecular chemical structure is shown in Figure 2.

### 3.2. Establishment of Molecular Model

**Figure 3:**
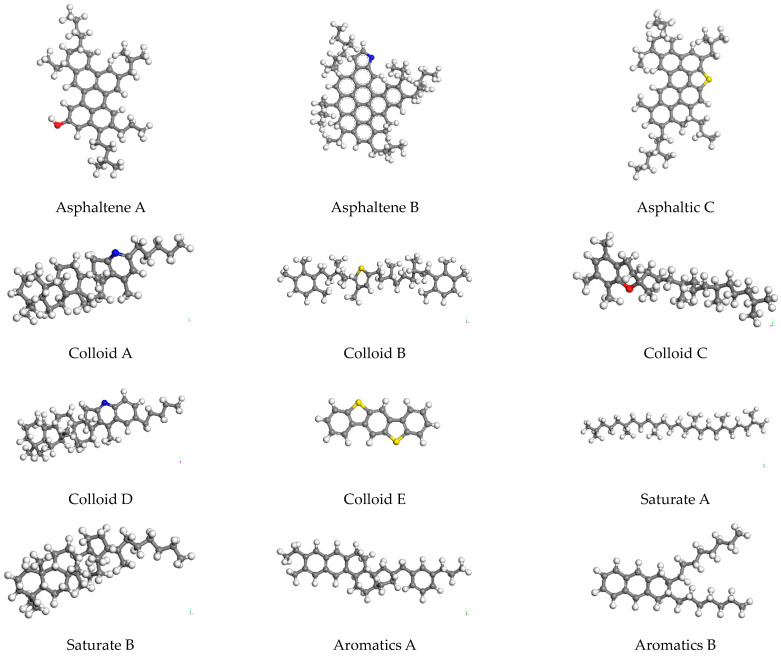
Molecular model diagram of asphalt components. (Note: in the model, red is atom O, blue is atom N, and yellow is atom S).

**Figure 4:**
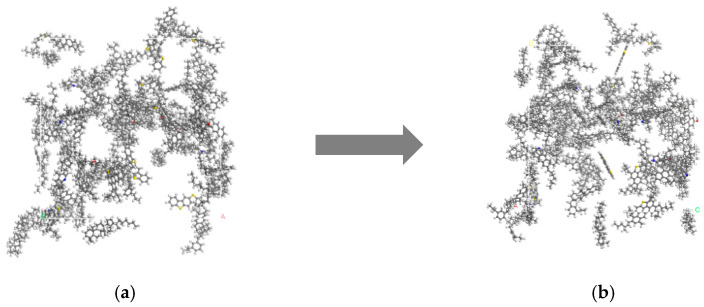
Model diagram of asphalt molecular structure (**a**) before optimization and (**b**) after optimization.

The establishment of an asphalt molecular model, based on MS 2020, used the Visualizer module to draw the molecular structures of asphalt components first, then added the corresponding number of molecular structures, and manually adjusted the number of each molecule used in model asphalt to adapt to the experimental data of asphalt morphology and atomic composition in SHRP asphalt. An amorphous unit cell was established in the z direction (i.e., vertical direction) as a contraction boundary condition. The molecular model of asphalt is complex, and the molecular dynamics algorithm itself had some limitations, which led to the obtained molecular structure of asphalt in not-the-most stable state. In order to eliminate the adverse reactions between atoms in the system and obtain the most stable molecular structure, it was necessary to minimize the energy utilization of the model to eliminate its influence before the molecular dynamics simulation. In this paper, structural optimization and annealing treatment were adopted for matrix asphalt molecules, and then the optimized and stable asphalt molecular model was obtained. The model was processed in the NVT regularization system and the NPT isothermal isobaric ensemble successively. After equilibrium pretreatment, the temperature range was set to 300 K~500 K, and the stress field was COMPASS. After annealing, the system energy tended to be stable, and a stable asphalt model was obtained, which eliminated and adjusted the unreasonable local structure in the original model and made the optimized model more compact. The molecular structure of 70# matrix asphalt 12 in MS is shown in Figure 3, and the molecular distribution of 70# matrix asphalt model is shown in Table 5. The molecular configuration of asphalt before and after optimization is shown in Figure 4.

## 4. Establishment of TiO_2_@LDO Model

### 4.1. TiO_2_ Model

**Figure 5:**
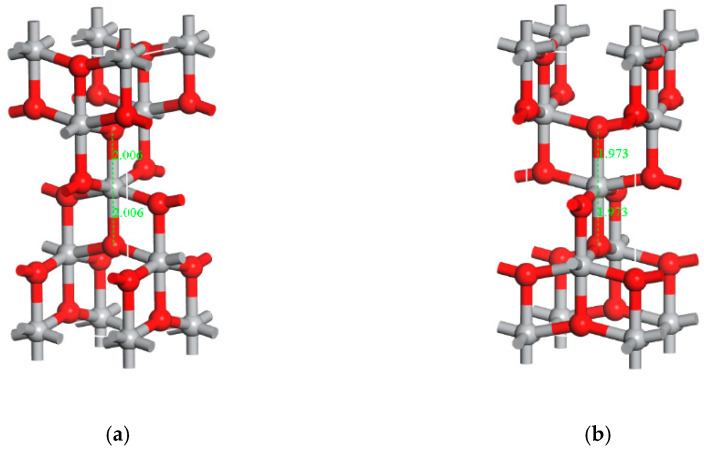
Model diagram before and after TiO_2_ optimization. The gray part is Ti atom and the red one is O atom. (**a**) before optimization and (**b**) after optimization.

There was an initial model of anatase TiO_2_ in the MS software library, so a model could be imported from it [35]. The CASTEP module in the software was used to configure the model with the lowest energy. The model before and after the treatment is shown in Figure 5. It can be seen that the bond length of the optimized Ti-O is changed from 2.006 to 1.973.

### 4.2. MgAl-LDHs Model

**Figure 6:**
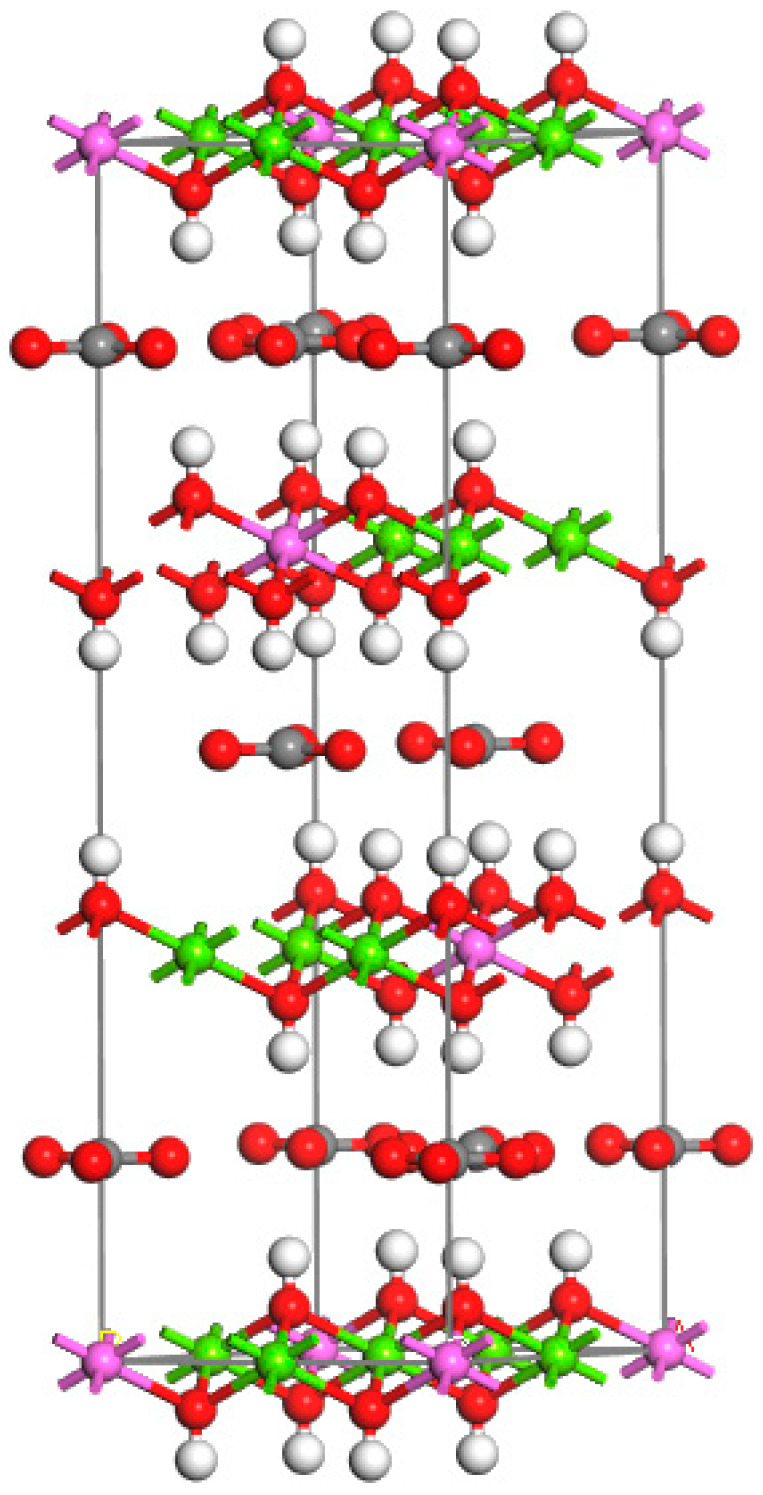
Model diagram after MgAl-LDHs optimization. The white part is hydrogen atom, the red one is oxygen atom, the pink part is aluminum atom, the green one is magnesium atom and the gray one is carbon atom.

For the MgAl-LDHs model, it was modelled directly in the software, and the BFGS algorithm, GGA-PBE/OTFG ultrasoft, and Density Mixing for geometric optimization were used. The optimized model is shown in Figure 6. 

### 4.3. TiO_2_@LDO Model

**Figure 7:**
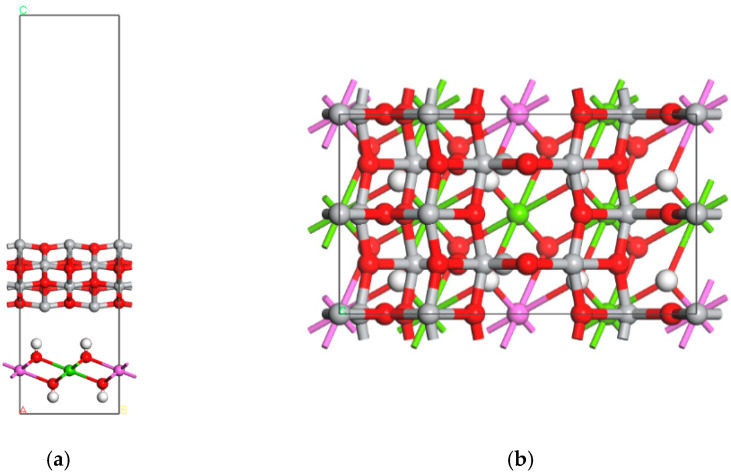
Model diagram of TiO_2_@ LDO composite material (**a**) front view and (**b**) top view.

For the above-mentioned optimized TiO_2_ and MgAl-LDHs models, the heterostructure was built, and the models were optimized. For specific steps, refer to Lin Bowen’s research [36]. The optimized MgAl-LDHs/TiO_2_ composite model, (i.e., TiO_2_@LDO), is shown in Figure 7.

## 5. Construction of Composite Photocatalytic Asphalt Model

In order to study the diffusion performance of asphalt and composite photocatalytic materials after compounding as well as the bonding performance between them, the heterostructure model of the above 70# matrix asphalt and TiO_2_@LDO composite was established and simulated in the software.

In the molecular dynamics calculation, viscosity was the macroscopic property of molecular weight, and in macromolecular solution, the viscosity of asphalt reflected solubility. In this paper, different temperatures are selected to study the model, and the simulated set temperatures were 0 °C, 20 °C, 40 °C, and 60 °C.

### 5.1. Bond Performance Analysis

The composite photocatalytic models were simulated at different temperatures by MS software, but it was difficult to compare the above-mentioned temperature models with naked eyes. In order to analyze the interaction energy between asphalt and composite photocatalytic materials with the increase in simulated temperature, it was necessary to introduce Einterface energy for more accurate evaluation and observation of the changing trend in the interaction energy.

The calculation formula of interface energy is as follows:

(1) $$\gamma_{\mathrm{int}}=\left(E_{polymer}+E_{surface}-E_{total}\right)/\left(2A\right)$$

In the formula,

$E_{polymer}$—crystal energy after polymer removal;

$E_{surface}$—polymer energy after crystal removal;

$E_{total}$—total energy of polymer and crystal;

A—interface area.

Because the interface area of the model is certain and the area is the same, the calculation formula of the interface energy can be simplified for the simulation of different temperatures, and the simplified formula is as follows:

(2) $$\gamma_{\mathrm{int}}=E_{polymer}+E_{surface}-E_{total}$$

We ran the Dynamic module through the Calculation of the Forcite module in the software to calculate the total energy $E_{\mathrm{total}}$, then removed the polymer and crystal in sequence, and calculated $E_{\mathrm{polymer}}$ and $E_{\mathrm{surface}}$ according to the above method, so as to obtain the interface energy at different temperatures. The calculation results are shown in Table 6.

According to existing research, when the interfacial energy is used to characterize the stability of the adsorption system, its numerical value shows a linear correlation trend with the interaction (i.e., adsorption). When the interfacial energy is zero or positive, it indicates that there is little interaction force or no adsorption phenomenon between them. If the interfacial energy is negative, it shows that there is interaction between them, and the larger the negative value, the stronger the adsorption capacity of both [37,38]. The greater the interface energy, the better the adhesion between asphalt and composite photocatalytic materials. As can be seen from Table 6, the interface energies are all negative, indicating that the interface between asphalt and TiO_2_@LDO has obvious adsorption. Among them, the interfacial energy followed a trend of 40 °C > 20 °C > 0 °C > 60 °C, and it can be seen that the interfacial energy between 40 °C and 60 °C is more obvious. From this, it can be concluded that the adsorption and bonding strength of asphalt and composite photocatalytic materials are the strongest at 40 °C.

### 5.2. Diffusion Performance Analysis

The diffusion performance between asphalt and composite photocatalytic materials can be analyzed from two angles, namely, particle movement and Z-direction density.

#### 5.2.1. Particle Motion Angle Analysis

The movement and migration of ions in space are the root causes of diffusion phenomena, so diffusion phenomena can be fundamentally analyzed by analyzing the motion of particles. However, due to the excessive number of particles in the software model, each particle had its own motion track, so it was impossible to analyze the specific motion of each particle. In the current research, MSD parameters were used to evaluate and analyze the particle diffusion, and the diffusion coefficient D could be calculated by one sixth of the slope of the diffusion stage curve.

The calculation formula of MSD parameters are as follows:

(3) $$MSD\left(t\right)=\langle {\left|r\left(t\right)-r\left(0\right)\right|}^{2}\rangle$$

In the formula,

〈〉—average all atoms in the group;

$r\left(t\right)$—position vector of particles in the group when time is t.

The formula of diffusion coefficient d is as follows:

(4) $$D=\lim\frac{1}{6t}MSD\left(t\right)$$

In the formula,

t—time.

**Figure 8:**
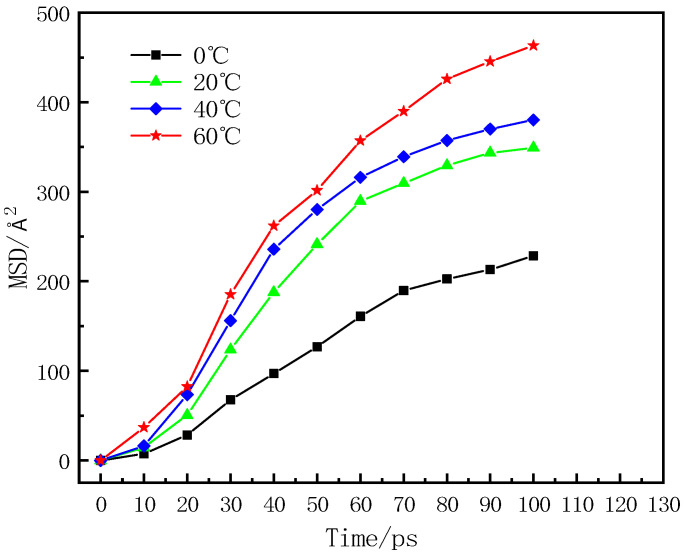
Curves of MSD with time at different simulated temperatures.

The calculation process of the MSD parameters was realized in the software. Specifically, the Dynamic module was carried out through the Analysis of Forcite module, and an xtd file was obtained. This file was analyzed, and the composite photocatalytic material TiO_2_@LDO was set as the set object. The specific analysis results are shown in Figure 8.

**Figure 9:**
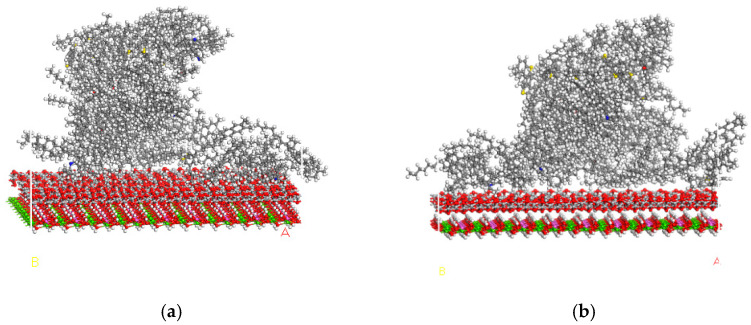

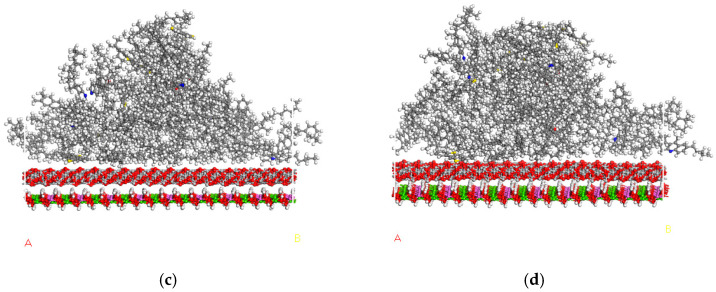
Interface model diagram under different simulated temperatures. (**a**) Interface diagram at 0°C, (**b**) interface diagram at 20 °C, (**c**) interface diagram at 40 °C, and (**d**) interface diagram at 60 °C.

As shown in Figure 8, the curve changed sharply during the time of 0 ps–60 ps, and the MSD parameter increased rapidly with time. The distance between asphalt and TiO_2_@LDO approached rapidly from far to near, and the change here is obvious. After a time of 60 ps, the MSD value tended to stabilize, meaning that the diffusion rate gradually stabilized. Meanwhile, the distance between asphalt and TiO_2_@LDO did not change much, and the bonding state between them tended to be stable. At this stage, there was no violent molecular movement, and atomic diffusion occurred at the interface between asphalt and TiO_2_@LDO, which was the stage of diffusion performance research. Figure 9 shows an interface model diagram at different simulated temperatures.

**Figure 10:**
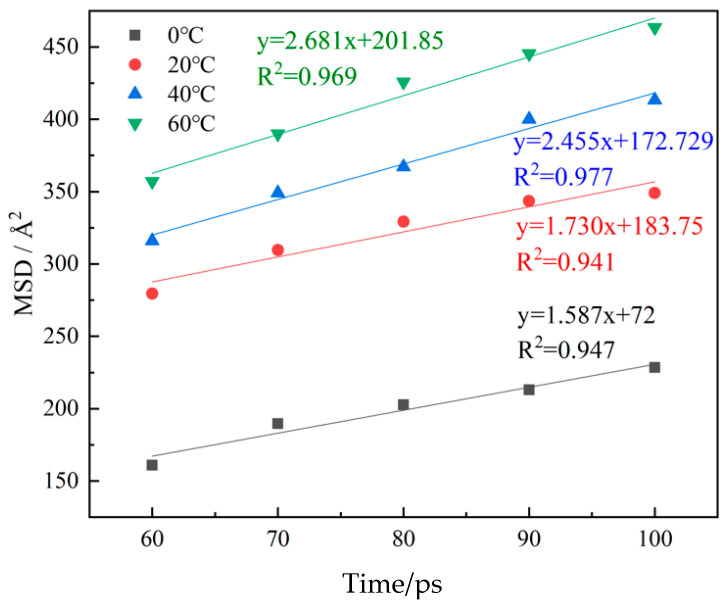
Linear fitting curves at different simulated temperatures.

Linear fitting of the MSD curve in the diffusion stage was used to obtain the slope sum of the fitted curve and $R^{2}$. Calculated through the diffusion coefficient formula, the fitting curve is shown in Figure 10, and the diffusion coefficient result is shown in Table 7.

It can be seen from Table 7 that the diffusion coefficient of TiO_2_@LDO was positively correlated with temperature, but it was not linear. The diffusion coefficient changes most obviously when the temperature changes from 20 °C to 40 °C, but the temperature had no obvious effect on the diffusion coefficient of TiO_2_@LDO after 40 °C.

#### 5.2.2. Density Analysis of TiO_2_@LDO along Z Direction

Because the MSD parameters can only reflect the motion activity of atoms, each atom has its directionality and its corresponding velocity in the process of diffusion motion, that is, the displacement in the displacement density distribution curve. In this paper, the change in the particle diffusion density of TiO_2_@LDO in the Z-direction was selected, and the particle displacement was analyzed in detail.

**Figure 11:**
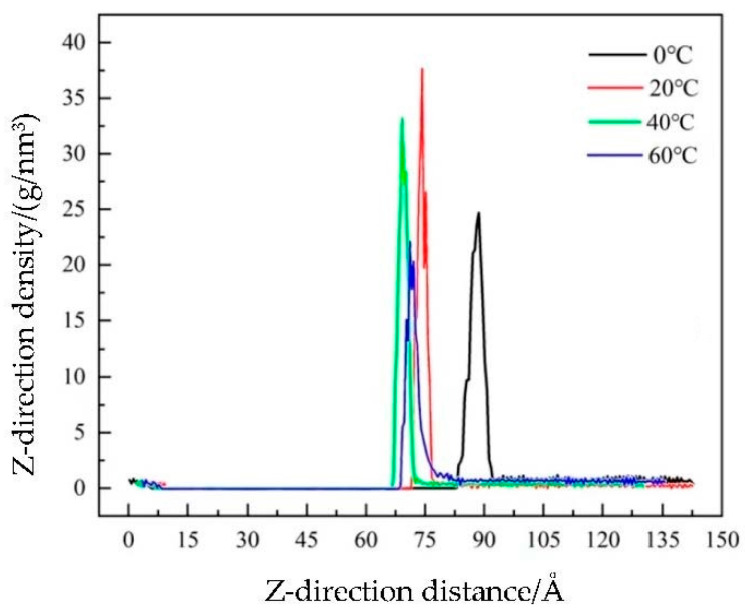
Density distribution of TiO_2_@LDO in different positions at different simulated temperatures.

The calculation process of the Z-direction density was realized in the software. Specifically, through the Concentration profile of the Analysis of Forcite module, an xtd file was obtained by running it dynamically, the file was analyzed, and the composite photocatalytic material TiO_2_@LDO was set as the set object to obtain the relative density data. The results were analyzed and the specific analysis results are shown in Figure 11.

As can be seen from Figure 10, two peaks, one high and one low, represent two TiO_2_@LDO layers. It was found that the distance between the two peaks gradually increased with the increase in temperature, and the density in the Z-direction also changed, which indicates that TiO_2_@LDO itself will not disperse with the increase in temperature, meaning that its structure is relatively stable. However, the diffusion between asphalt and TiO_2_@LDO was stronger with the increase in temperature, and the interactive bonding reaction between asphalt and TiO_2_@LDO was stronger, which provides a theoretical basis for TiO_2_@LDO to modify matrix asphalt.

## 6. Conclusions and Discussion

In this paper, firstly, an asphalt model of SHRP AAA-1 based on SARA was constructed by MS. The molecular structure of the four-component 12 of 70# base asphalt included two molecular components of saturated component, two molecular components of aromatic component, three molecular components of asphaltene, and five molecular components of gum. The characteristic values of asphalt four-component molecular structure, and the molecular distribution ratio of each component in the molecular model, were given. Then, the optimized TiO_2_ and MgAl-LDHs models were constructed with heterostructures, and the TiO_2_@LDO composite model was established. Next, the heterostructure model of the above 70# matrix asphalt and TiO_2_@LDO composite materials was established, and the bonding performance between the asphalt and composite photocatalytic materials was analyzed by interface energization. Additionally, the diffusion performance between asphalt and composite photocatalytic materials was analyzed from the perspective of particle movement and Z-direction density, drawing the following conclusions.

(1) TiO_2_@LDO molecules have certain a attraction to asphalt molecules, so TiO_2_@ LDO can be better combined with asphalt to form a heterostructure, and matrix asphalt can be modified by TiO_2_@LDO.

(2) In the temperature range of 0 °C~60 °C, the interfacial energy between asphalt -TiO_2_@LDO is 40 °C > 20 °C > 0 °C > 60 °C, and the adsorption and bonding strength between asphalt and TiO_2_@LDO are the strongest at 40 °C.

(3) In the process of TiO_2_@LDO diffusion, TiO_2_@LDO diffuses obviously on the surface of asphalt molecules. With the increase in simulated temperature, the larger the diffusion coefficient of TiO_2_@LDO is and the more active the movement of TiO_2_@LDO is.

(4) The density analysis of TiO_2_@LDO along the Z direction shows that TiO_2_@LDO molecules diffuse more widely at 60 °C, which lays the foundation for further blending of asphalt and TiO_2_@LDO.

(5) With the increase in simulated temperature, the higher the diffusion coefficient of TiO_2_@LDO is and the better its high-temperature performance can be. This is because as the temperature increases, TiO_2_@LDO molecules diffuse more into asphalt molecules and further blend with them, which also provides a theoretical basis for the influence of temperature on photocatalytic efficiency.

The innovation of this paper was to introduce TiO_2_@LDO into asphalt material, so that it displayed photocatalytic performance and adsorption performance at the same time. Subsequently, the composite photocatalytic asphalt material could be used in road engineering to improve degrade automobile exhaust and improve the air environment in the road area.

Due to time constraints, at present, this paper only studied the properties of composite photocatalytic asphalt materials from the theoretical simulation level. In follow-up research, further investigation is needed on the following aspects.

(1) In this paper, only the molecular dynamics simulation of matrix asphalt was carried out, and the related research of modified asphalt, such as SBS asphalt, could be further explored.

(2) This paper only carries out theoretical analysis. As such, relevant experimental research could be carried out on the influence of composite photocatalytic materials on the road performance of asphalt materials, as well as further theoretical-experimental interactive analysis.

## Figures and Tables

**Table 1:** Basic performance parameters of 70 # asphalt.

Technical Indicators		Unit	Technical Requirement	Test Result
Penetration (25 °C, 100 g, 5 s)		0.01 mm	60~80	67.5
Ductility (10 °C, 5 cm/min)		cm	≮15	31.8
Softening point		°C	≮46	52.5
Viscosity (135 °C)		—	-	484.5
Flash(ing) point		°C	≮260	310
Wax content (distillation method)		%	≯2.2	1.21
Density (15°C)		g/cm^3^	Measured record	1.035
Solubility (trichloroethylene)		%	≮99.5	99.88
Thin film heating test 163 °C, 5 h	Mass loss	%	≯±0.8	−0.11
	Penetration ratio at 25 °C	%	≮61	65.64
	Ductility (10 °C)	cm	≮15	22.7

**Table 2:** Basic performance parameters of nano-TiO_2_.

Technical Indicators	Unit	Technical Requirement	Test Result
Exterior	—	White powder	White powder
Average grain diameter	nm	10 ± 5	10.3
PH value of aqueous suspension	—	6~7	6.8
Apparent density	—	≯0.30	0.28
Titanium dioxide content	%	≮99.5	99.9
Specific surface area	m^2^/g	≮120	124
Drying shrinkage	%	≯0.5	0.45
Burning weightlessness	%	≯1.0	0.89
Moisture	%	≯1.5	1.3

**Table 3:** Basic performance parameters of LDHs.

Technical Indicators	Unit	Technical Requirement	Test Result
Exterior	—	White powder	White powder
Density (25 °C)	g/cm^3^	2	2
Melting point	°C	>300	325
Flash point	°C	>110	115
Mg-Al hydrotalcite content	%	≮99.4	99.9
Boiling point (760 mmHg)	°C	333.6	333.6

**Table 4:** Eigenvalues of four-component molecular structure of asphalt.

SARA Component	Molecule	Molecular Name	Structural Formula	Relative Score Submass/g mol^−1^	Atomicity	Code
Saturate	Saturate A	Squalance	C_30_H_62_	422.9	92	a
	Saturate B	Hopane	C_35_H_62_	483.0	97	b
Aromatics	Aromatics A	PHPN	C_35_H_44_	464.8	79	c
	Aromatics B	DOCHN	C_30_H_46_	406.8	76	d
Asphaltene	Asphaltene A	Asphaltene-phenol	C_42_H_54_O	575.0	97	e
	Asphaltene B	Asphaltene-pyrrole	C_66_H_81_N	888.5	148	f
	Asphaltene C	Asphaltene-thiophene	C_51_H_62_S	707.2	114	g
Colloid	Colloid A	Pyridinohopane	C_36_H_57_N	503.9	94	h
	Colloid B	Thioisorenieratane	C_40_H_60_S	573.1	101	i
	Colloid C	Trimethylbenzene-oxane	C_29_H_50_O	414.8	80	j
	Colloid D	Quinolinohopane	C_40_H_59_N	554.0	100	k
	Colloid E	Benzobisbenzothiophene	C_18_H_10_S_2_	290.4	30	l

**Table 5:** Molecular distribution ratio of components in molecular model of 70 # base asphalt.

SARA Component	Molecule	Relative Molecular Mass/g mol^−1^	Atomicity	Number of Model Molecules	Model Atomic Number	Mass Fraction (%)	Model Scale (%)
Saturate	A	422.9	92	5	460	7.3	15.6
	B	483.0	97	5	485	8.3	
Aromatics	A	464.8	79	15	1185	24.1	45.2
	B	406.8	76	15	1140	21.1	
Asphaltene	A	575.0	97	2	194	4.0	15.0
	B	888.5	148	2	296	6.1	
	C	707.2	114	2	228	4.9	
Colloid	A	503.9	94	3	282	5.2	24.2
	B	573.1	101	3	303	5.9	
	C	414.8	80	3	240	4.3	
	D	554.0	100	3	300	5.7	
	E	290.4	30	3	90	3.0	

**Table 6:** Interface energy of asphalt-composite photocatalytic materials.

Simulated Temperature/°C	*E_total_*/kcal·mol^−1^	*E_polymer_*/kcal·mol^−1^	*E_surface_*/kcal·mol^−1^	*E_interface_*/kcal·mol^−1^
0	302,161	3388	298,882	−109
20	302,089	3335	298,879	−124
40	302,102	3344	298,885	−127
60	302,131	3360	289,977	−107

**Table 7:** Diffusion coefficient of TiO_2_@LDO at different simulated temperatures.

Temperature/°C	Diffusion Coefficient/()×10^−7^$\;m^{2}/s$	$R^{2}$
0	2.645	0.947
20	2.883	0.941
40	4.075	0.977
60	4.468	0.969

## Data Availability

Data will be made available on request.
